# Gene flow of common ash (*Fraxinus excelsior* L.) in a fragmented landscape

**DOI:** 10.1371/journal.pone.0186757

**Published:** 2017-10-20

**Authors:** Devrim Semizer-Cuming, Erik Dahl Kjær, Reiner Finkeldey

**Affiliations:** 1 Department of Forest Genetics and Forest Tree Breeding, Faculty of Forest Sciences and Forest Ecology, University of Göttingen, Göttingen, Germany; 2 Department of Geosciences and Natural Resource Management, University of Copenhagen, Copenhagen, Denmark; Consiglio Nazionale delle Ricerche, ITALY

## Abstract

Gene flow dynamics of common ash (*Fraxinus excelsior* L.) is affected by several human activities in Central Europe, including habitat fragmentation, agroforestry expansion, controlled and uncontrolled transfer of reproductive material, and a recently introduced emerging infectious disease, ash dieback, caused by *Hymenoscyphus fraxineus*. Habitat fragmentation may alter genetic connectivity and effective population size, leading to loss of genetic diversity and increased inbreeding in ash populations. Gene flow from cultivated trees in landscapes close to their native counterparts may also influence the adaptability of future generations. The devastating effects of ash dieback have already been observed in both natural and managed populations in continental Europe. However, potential long-term effects of genetic bottlenecks depend on gene flow across fragmented landscapes. For this reason, we studied the genetic connectivity of ash trees in an isolated forest patch of a fragmented landscape in Rösenbeck, Germany. We applied two approaches to parentage analysis to estimate gene flow patterns at the study site. We specifically investigated the presence of background pollination at the landscape level and the degree of genetic isolation between native and cultivated trees. Local meteorological data was utilized to understand the effect of wind on the pollen and seed dispersal patterns. Gender information of the adult trees was considered for calculating the dispersal distances. We found that the majority of the studied seeds (55–64%) and seedlings (75–98%) in the forest patch were fathered and mothered by the trees within the same patch. However, we determined a considerable amount of pollen flow (26–45%) from outside of the study site, representing background pollination at the landscape level. Limited pollen flow was observed from neighbouring cultivated trees (2%). Both pollen and seeds were dispersed in all directions in accordance with the local wind directions. Whereas there was no positive correlation between pollen dispersal distance and wind speed, the correlation between seed dispersal distance and wind speed was significant (0.71, p < 0.001), indicating that strong wind favours long-distance dispersal of ash seeds. Finally, we discussed the implications of establishing gene conservation stands and the use of enrichment planting in the face of ash dieback.

## Introduction

Pollen and seed dispersal patterns are important to determine the consequences of ecological and biological threats on plant populations, such as landscape changes, climate change and newly emerging pathogens. Habitat fragmentation can reduce genetic connectivity of plant populations [1] by reducing habitat size and increasing spatial isolation. The reduced population sizes over generations can decrease genetic variation within populations while increasing genetic differentiation among populations due to increased random genetic drift, inbreeding and restricted gene flow [2]. However, depending on the differences in species’ responses, habitat fragmentation can also facilitate gene flow among fragmented populations [3–6]. It is therefore important to improve the understanding of gene flow patterns in both fragmented and non-fragmented forest ecosystems [7].

Natural forests are often replaced with managed large-scale monocultures, in order to meet the demand for wood and other forest products [8]. The global area of planted forests increased from 167.5 to 277.9 million ha between 1990 and 2015, and the increase was the second most rapid in Europe (37%) [9]. By 2015, the planted forest area in Germany increased to 5.2 million ha with an annual increase of 2800 ha from 1990 to 2000 [9]. Such forest activities often involve the introduction of new genetic variants, and gene flow from plantations can influence the genetic diversity of native populations. If the gene flow from plantations is pronounced, it may result in loss of genetic diversity that reflects adaptation to unique ecological conditions and therefore call for specific gene conservation efforts [10,11]. On the other hand, allochthonous gene flow from plantations might prevent genetic erosion of small native populations by restoring genetic variation and reducing inbreeding depression, leading to a recovery of fitness or genetic rescue [12,13]. However, the actual degree of gene flow from plantations to old growth forests is unknown for most plantation programs.

Emerging infectious diseases (EIDs) are another potential cause behind loss of genetic diversity and local genetic patterns. Increasing in number worldwide [14], EIDs are typically introduced by humans to new ecosystems where they have not coevolved with their new host. They can threaten biodiversity through biomass loss and/or extinction of host species [15]. There are several examples of EIDs that have led to dramatic mortality in previously widespread tree species, but trees with natural resistance are often found in low frequency in natural populations [16]. Offspring of such rare survivors could found future populations. Accurate estimation of pollen and seed dispersal patterns in natural populations is therefore fundamental to predict the response of forest tree populations and ecosystems to such threats, since gene dispersal capacity will determine how fast high-fitness genes can increase in frequency across landscapes, and thereby the recovery potential of the species. In the absence of genetic connectivity, mating among related trees in genetic clusters around surviving trees can lead to inbreeding. Gene conservation strategies, in general, aim at creating high population connectivity among local gene pools while limiting unwanted gene flow [1,10,17]. In the case of EIDs this is complicated by the need for dispersal of resistance genes without losing important patterns of local adaptation.

### Dieback and gene flow in ash

Common ash (*Fraxinus excelsior*, hereinafter ash), is a keystone species in natural plant communities in European forests [18]. It is distributed across Europe, from the Atlantic coast in the west to continental Russia almost up to the River Volga in the east, from Norway in the north to the northern parts of Spain, Italy, Greece, and as far as Iran in the south [19]. Ash is a valuable tree species in terms of its ecological characteristics, wood quality and high economic value [20]. However, the species is highly threatened by a pathogenic fungus, *Hymenoscyphus fraxineus* (synonym *H*. *pseudoalbidus*, anamorph *Chalara fraxinea*), which causes dieback [21–23]. The disease symptoms were observed in Poland in the early 1990s [24], but has since spread throughout Europe, causing massive tree mortality and threatening the species’ existence in many forest ecosystems [25–27]. Dieback symptoms were first observed in Northern Germany in 2002, and since then increased damage has been reported throughout the country, resulting in ecological and economic impacts on forests, open landscapes, nurseries and urban plantings [28]. Previous studies have identified high genetic variation in disease susceptibility levels, indicating the species’ genetic potential to recover through natural and artificial selection [29–31]. However, the speed of pathogen spread is expected to be much faster than the spread of resistance at the population level, because the frequency of tolerant trees is low [27] and ash trees rarely flower before 10 years old. As a result, the speed of population recovery will depend on the survival rate, the reproductive fitness of surviving trees and the extent of pollen- and seed-mediated gene flow.

### Estimation of gene flow based on parentage analysis

Categorical approach to parentage analysis [32] assigns candidate parents to their offspring at certain confidence levels using genetic information and thereby makes it possible to determine the distances and directions of realized pollen and seed dispersal events. The neighbourhood model [33–35], on the other hand, estimates mating model parameters, directly accounting for spatial distribution of individuals as well as their phenotypes, and infers genealogies after fitting the parameters. Parentage analysis allows detailed reconstruction of ongoing mating patterns. This may be used to precisely locate individuals or their groups with low versus high reproductive success. The neighbourhood model, however, allows accounting for confounding effects of dispersal and fecundity, and provides a predictive tool (the forward dispersal kernel [36]) for seed and pollen dispersal. Such a model can be particularly useful in the assessment of gene flow potential of a species. Therefore, using a combination of these approaches can provide more information on gene flow estimations.

Pollen and seed dispersal distances and directions can be calculated once paternal (pollen) and maternal (ovule-seed) contributions are identified. With seeds sampled on specific mother trees, pollination distances and directions can easily be calculated. However, in the case of seedlings, additional information regarding the gender of the both candidate parents is required. A prior assumption of the closest parent being the maternal parent is potentially misleading and can underestimate the seed dispersal distances [37]. Uniparentally inherited cytoplasmic markers can help revealing seed and pollen parents [38]. In the case of angiosperms, however, polymorphism levels at regional scales are often low with a single haplotype dominating in most regions [39]. It is therefore valuable to use repetitive macroscopic observation of ash flowers to identify gender.

The process of propagule dispersal is complex, and is influenced by both biotic and abiotic factors. For wind-mediated dispersal, wind conditions are obviously expected to play a substantial role in determining the dispersal directions and distances from parent trees. For example, dry, windy weather conditions promote long-distance dispersal (LDD) of seeds [40–43]. Likewise, clear warm days with low relative humidity, especially from midday to late afternoon (10 am-6 pm), are most favourable for pollen release [44–46]. Parentage assignment methods allow tracking of pollen and seed dispersal events in terms of distance and directionality [47]. Additional meteorological data could help explain the potential range of pollen and seed dispersals within particular landscapes, regions or continents [48], and enable us to better understand to what extent these factors affect the dispersal patterns.

### Objectives of the present study

The aim of the present study is to investigate genetic connectivity among ash trees in a fragmented landscape in Rösenbeck, Germany. A combination of categorical parentage and the neighbourhood model approaches was applied to estimate mating parameters and to describe dispersal patterns in our study site. Specifically, we studied the degree of reproductive isolation of a 2 ha natural population from cultivated trees planted within a few hundred meters. Gender information was used to calculate pollen and seed dispersal distances and directions. Actual wind and precipitation data were utilized to assess the effect of wind on the dispersal patterns. The outcomes are discussed in relation to the genetic management of ash, including its conservation and reproductive material use, especially under the current epidemic ash decline.

## Materials and methods

### Study species reproductive ecology

Both pollen and seed dispersal are mediated by wind in *F*. *excelsior*. Large numbers of female flowers are produced and pollinated in spring, before leaf flush. The pollination season in Northern and Central Europe lasts from April to May [49]. Seeds mature in September- October and the majority of the seeds are dispersed between October and February [50]. A fraction of the seeds can remain on the trees until the following summer [51]. The dispersal phenology corresponds to a deep seed dormancy that is not released until seeds have experienced both warm and cold stratification. The majority of the seeds, therefore, do not germinate until the second year after maturation [52].

The species presents a polygamous reproductive system where the individuals are phenotypically classified in a continuum from purely male to purely female, with a wide range of hermaphroditic intermediates [53,54], but it is regarded as functionally dioecious [55]. Although hermaphrodite flowers are self-fertile, their paternal success is much lower than that of males at the seed set stage [56]. Earlier release of male pollen outcompetes hermaphrodite pollen in cross-pollinations, suggesting hermaphrodite flowers function predominantly as female [57]. High daily concentrations of ash pollen (> 100 pollen grain/m^3^) can remain in the air between 10–20 days [58].

### Study site, demography of ash and sampling design

**Fig 1:**
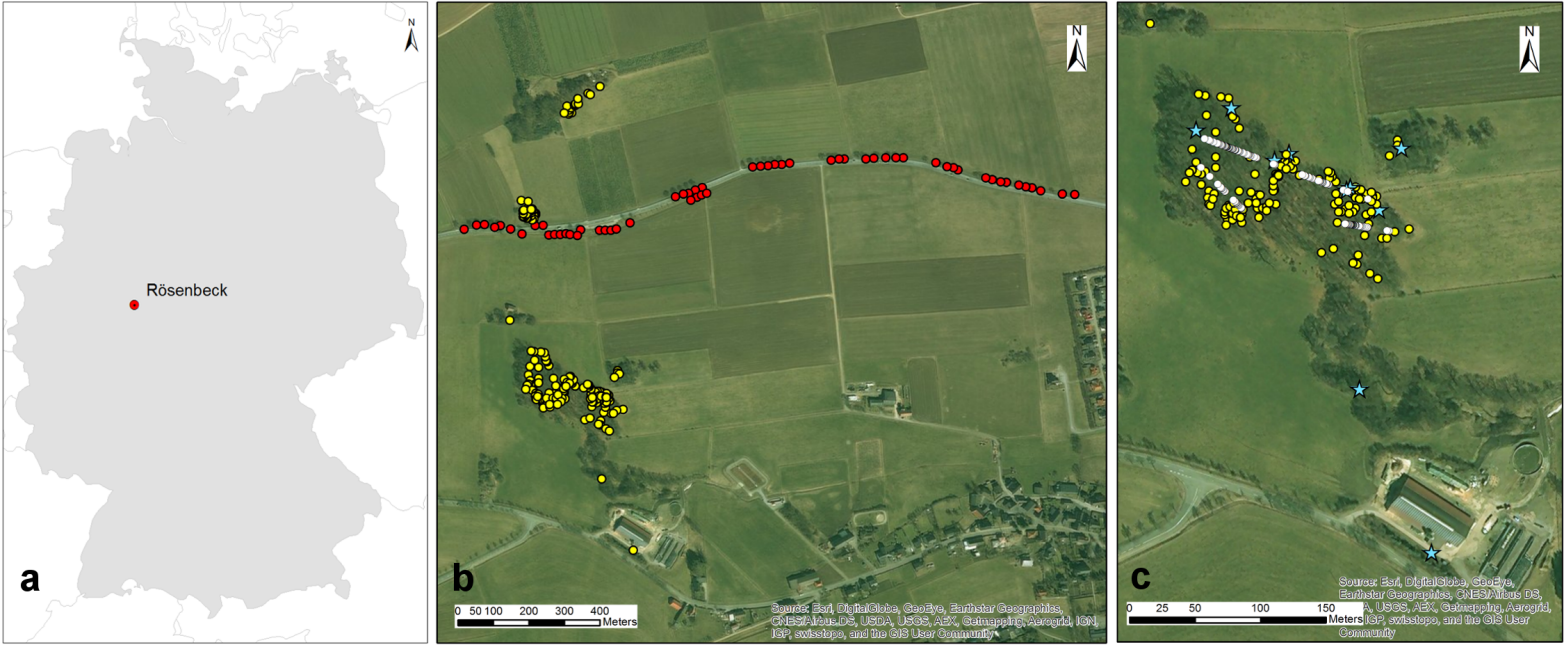
Map of the study site and sampling scheme. (a) Location of the study site in Germany. (b) Sampling of the adult trees (cultivated trees along the road are shown as red circles and native trees in the landscape are marked as yellow circles). (c) Sampling of the offspring (mother trees of the seeds were illustrated as blue stars and the seedlings collected along 3 transects were marked as white circles). The maps were created using ArcMap™ 10.4.1, Copyright © ESRI.

The location of the study site and the sampling scheme are shown in Fig 1. The study site is located about 1 km northwest of Rösenbeck (51°24'36" N 8°40'12" E), Brilon, North Rhine-Westphalia, Germany (Fig 1A). The fragmented landscape is composed of agricultural lands, forest patches and a highway spanning the middle. The oldest available map (1816–1850) of Brilon (www.tim-online.nrw.de/tim-online/initParams.do) revealed the historical fragmentation of the region, indicating that the landscape fragmentation must be at least 200 years old. In a radius of 5 km around the study site, ash mainly exists as single trees in different stands, mostly mixed with beech, and only exists seldom in more or less small pure tands. Thus the estimated abundance of ash in the area is less than 5% (communicated with Martin Rogge from Arnsberg Forest, Landesbetrieb Wald und Holz Nordrhein-Westfalen).

The cultivated alley trees stretch for 1 km along both sides of the highway (Fig 1B). Apical and terminal buds were sampled from all mature, reproductive trees in the landscape (for about 500 m from the boundaries of the native stand) in January 2014 (Fig 1B). Among 268 adult trees sampled, 58 of them were alley trees. According to our estimations, the age of the native trees ranged from 20 to 80, with a few additional older ones, about 100–120 years old. The deciduous tree symbols placed on the historical map, covering the time period of 1936 and 1945 may indicate the time of establishment of the native forest, supporting our age estimations. Offspring were sampled in the privately owned native forest (2 ha) (Fig 1C). Leaves were sampled from 247 seedlings in July in the same year along 3 main transects, representing different parts of the forest (Fig 1C). A total of 500 seeds were collected on 10 mother trees (50 seeds per mother) in October 2014. Two of the mother trees are marginal; one of them is located in the southernmost edge of the forest while the other one is located in the land owner’s factory yard (Fig 1C).

### Field data collection

The geographical position of each adult tree was recorded during the sampling period using Garmin GPSmap® 62s. The position of each seedling on the transect line relative to the start point was recorded, and the heights of the seedlings were measured. Visual observation of the adult tree flowers was conducted in two subsequent years in 2014 and 2015 when both cultivated and native trees were flowering at the same time. The flowers were observed between 11^th^ -17^th^ of April in 2014 and 21^st^ -22^nd^ of April in 2015 because flowering started earlier in 2014 relative to 2015. The trees were recorded as male, female or hermaphrodite according to their flower types. In case of the absence of flowering, any additional observations informative for gender identification, such as presence of dry seeds and seed stalks from the previous year and flower galls [59], were recorded. Seed set was observed in 2014 and 2015, in August.

### DNA extraction and microsatellite analysis

Leaves and buds were stored at -20°C until they were used for DNA extraction. Seeds were collected and stored at 4°C in labelled bags. Prior to embryo extraction, randomly picked seeds were embedded in water for 1–2 days after pericarp tissues were removed. After the rehydration, seeds were sliced into two halves with a razor blade and embryos were carefully picked up to avoid any contamination with maternal tissues. Total DNA was extracted from leaf, bud and embryo tissues using DNeasy® 96 Plant Kit (Cat No. 69181) according to the manufacturer’s protocol (Qiagen, Hilden, Germany).

Microsatellite analyses were performed using 13 primers. FEMSATL8, FEMSATL11 and FEMSATL19 are frequently used primers developed for *F*. *excelsior* [60]. ASH2429 [61], FRESTSSR279, FRESTSSR308, FRESTSSR427, FRESTSSR528 [62] and Fp12378, Fp18437, Fp20456, Fp21064, Fp104136 [63] are EST-SSRs developed for *F*. *excelsior* or other *Fraxinus* species. The primers were labelled with two different fluorescent dyes (6[FAM]: FEMSATL8, FEMSATL19, ASH2429, FRESTSSR308 and Fp18437, Fp21064, Fp104136; 6[HEX]: FEMSATL11, FRESTSSR279, FRESTSSR427, FRESTSSR528 and Fp12378, Fp20456), and were combined in 4 Multiplexes (M) (M1: FEMSATL11, Fp18437 and Fp21064; M2: FEMSATL19, FRESTSSR427 and Fp12378; M3: FEMSATL8 and ASH2429, M4: FRESTSSR279, FRESTSSR308, FRESTSSR528, Fp20456 and Fp104136). Amplifications were performed in 14 μl reactions. Each reaction contained 1.5 μl of 1× Reaction Buffer: 0.8 M Tris-HCl (pH 9.0), 0.2 M (NH_4_)_2_SO_4_, 0.2% w/v Tween-20 (Solis BioDyne, Tartu, Estonia), 2.5 mM MgCl_2_, 0.2 mM dNTP mix, 0.2 μl 1 U of Taq DNA polymerase (HOT FIREPol® DNA Polymerase, Solis BioDyne, Tartu, Estonia), and 0.3 μM forward and reverse primer, and ~ 10 ng genomic DNA. A combination of hot start and touchdown procedure was applied for PCR conditions. Thermal cycling conditions were as follows: 95°C for 15 min followed by 10 cycles of 94°C for 1 min, annealing temperature step downs each cycle of 1°C (from 60°C to 50°C) for 1 min, 72°C for 1 min. The annealing temperature for the final 25 cycles was 50°C for 1 min with the denaturation and extension steps as above. The final extension step was at 72°C for 20 min. PCR products were analyzed using Applied Biosystems 3130xl Genetic Analyzer (Applied Biosystems, Foster City, CA, USA). Allele size calling was carried out with GeneMapper 4.1^®^ (Applied Biosystems, Foster City, USA) using the software developer’s default settings for the analysis parameters, except for the parameters for allele peak quality. Signal levels for minimum homozygous and heterozygous peak height were set to 200 RFU and 100 RFU, respectively. Minimum peak height ratio was set to 0.5 for heterozygote balance. Each individual genotype was checked for errors, and the analyses were repeated for missing or uncertain peaks. Mother-offspring mismatches in all loci were checked and repeated when necessary to avoid scoring PCR artefacts because of null alleles. Manual allele binning was conducted based on the microsatellite repeat units and the cumulative allele size distributions were plotted in Excel for each locus. Manually binned alleles were compared to the ones generated by TANDEM, an automated allele binning software [64], to detect potential allele binning errors.

### Population genetics analysis

Genetic diversity parameters including number of alleles (A), effective number of alleles (A_e_), observed heterozygosity (H_o_), Hardy-Weinberg expected heterozygosity (H_e_) of cultivated and native adults and offspring were calculated using GENALEX 6.5 [65]. Allelic richness (A_R_) was computed using rarefaction method in HP-RARE [66] to avoid bias due to different sampling size. INEST 2.1 software [67] was used for unbiased multilocus estimates of inbreeding coefficients within population (F_IS_). The software jointly estimated inbreeding coefficients and null allele rates according to the Bayesian approach at the parameter settings of 200 000, 2000 and 20 000 for the number of cycles, thinning and burnin, respectively. Pairwise genetic differentiations (F_ST_) and their significance were determined based on 9999 permutations using GENALEX 6.5 [65]. Each locus was checked for presence of stuttering and large allele drop-out using MICRO-CHECKER 2.2.3 [68].

### Parentage analysis

The assessment of gene flow in our study site was carried out with two approaches. The categorical approach to parentage assignment [69] was applied for direct identification of the most likely parents of the offspring (seeds and seedlings), and to estimate pollen and seed dispersal distances and directions from the parents to the location of the propagule deposition. The spatially explicit neighbourhood model approach [33–35] was additionally applied to provide more accurate estimates for the proportion of seed and pollen immigration and to further assess the patterns of parentages when accounting for spatial distributions of the individuals in our study site.

#### Categorical approach

Preliminary microsatellite analysis revealed the presence of identical genotypes among the cultivated trees, indicating their vegetative propagation in the past. Among 58 cultivated trees, 36 of them presented one single genotype (Genotype 1) while 15 of them presented another (Genotype 2) (S1 Table). Only one representative genotype from each group was kept for further analyses to prevent interferences with the parentage assignments due to identical log-likelihood (LOD) scores. A total of 9 different genotypes (Genotype 1 + Genotype 2 + 7 unique genotypes) represented the cultivated tree gene pool in the parentage analyses.

Parentage assignments were performed using CERVUS 3.0.7 [70]. Parent pair analysis assigned candidate parent pairs to the seedlings, while paternity analysis assigned candidate fathers to the seeds with known mothers. Parentage assignments were made based on Delta (Δ), the difference in LOD scores between two most likely parents, in order to increase the certainty of the identity of true parents where multiple parents had positive LOD scores [69]. Single parent assignments were performed based on their LOD scores computed in the parent pair analysis. Prior to the assignments, simulations were run to calculate the critical values of LOD and Δ at strict (95%) and relaxed (80%) confidence levels. Because there were no missing data in the genotype data set, the minimum number of loci was set to 13. The default value of 1% was kept as the proportion of error rate in the simulations. Selfing was incorporated into the simulations owing to the self-fertilization ability of ash hermaphrodites. The number of simulated offspring was set to 100 000 to increase the reliability of the critical LOD values. In spite of the complete sampling scheme of the study, the parameter for percentage of sampled potential parents was set to 85% for the parent pair analysis due to the significant potential of long-distance gene flow of ash in fragmented landscapes. The same parameter was lowered to 75% for the paternity analysis since 20% of the seeds were collected from the mother trees in the margin of the forest patch.

After the parentage analyses, each confident assignment was subjected to further elimination using the gender information of each adult tree acquired from field observations in 2014 and 2015, in order to improve the reliability of the assignments. The assignments were excluded from further calculations when there was a discrepancy between the genders of the two assigned parents or no information about the genders of the assigned parents. This was only the case for 37 (out of 332) paternity, and 11 (out of 121) parent pair assignments. The confidently assigned single parent was only accepted as a seed parent when it was female and having no mismatching loci with the seedling. This was the case for 23 (out of 67) of the assigned single parents.

Point coordinates (*x*, *y*) of the seedlings relative to their respective transect lines were calculated using Euclidean distances. These coordinates were later transformed into the same geospatial coordinates with the adult trees to calculate the pollen and seed dispersal distances. To identify the directions of the dispersal events, azimuths (horizontal angle measured clockwise from the North) were calculated using QGIS 2.16 [71]. Pollen dispersal directions were calculated from father to mother tree while seed dispersal directions were calculated from mother tree to seedling. A clonal genotype was assigned as a parent in 12 cases in total. In this case, mean pollination and seed dispersal distances and angles were taken into account.

#### The neighbourhood model approach

NMπ, a successor of NM+ software [72] was used to characterize mating/dispersal patterns in our study population based on the neighbourhood model. The estimated model parameters were frequency of self-fertilization, frequency of seed and pollen immigration from surrounding sources, mean distance of seed and pollen dispersal, shape of seed and pollen dispersal kernels, intensity (rate) of directionality (anisotropy) of seed and pollen dispersal and prevailing direction of seed and pollen dispersal. The software also estimated per locus genotyping error rates. The genders of the adult trees were incorporated into the model estimations according to their femaleness where 0 stands for male, 0.5 stands for hermaphrodite or unknown sex and 1 stands for female. A stepwise approach was followed during the estimations due to the sensitivity of the model estimations to initial input values. At first, genotyping error rates, frequency of immigrations and selfing rates were estimated. Using these parameters, four models were applied to estimate average dispersal distances and rate of directionalities based on exponential-power kernel. Simple exponential kernel (b = 1), fat-tailed (b < 1) and normal (b = 2) distributions were tested in these models. The model comparisons were made based on the Akaike Information Criterion (AIC) [73], and the best-fitting model was selected according to the calculated relative weights (e.g. [74]). The two most-likely genealogies inferred for each offspring in the best-fitting model were evaluated according to their associated posterior probabilities. The minimum threshold probability of 0.8 was taken into account to evaluate the seed and pollen dispersal distances in the study site. The dispersal potential in the study site was assessed based on the estimated fitted dispersal kernel function. The approximate 95% confidence interval around dispersal kernel parameters, mean distance of seed and pollen dispersal and shape of seed and pollen dispersal kernels were estimated. Based on the best-fitting dispersal kernels, predictive cumulative probability distributions were computed using NM+ software.

### Effect of wind on dispersal patterns

To assess the effects of wind direction and speed on the realized dispersal events, publicly available meteorological data was acquired from Climate Data Centre of German National Meteorological Service (ftp://ftp-cdc.dwd.de/pub/CDC/observations_germany/climate/hourly/) (S2 Table). Based on the flower observations in the study site in 2014, the time period within 10^th^-25^th^ of April was considered as pollination period. Accordingly, hourly wind data in the same period in April 2014 was extracted from Haaren Station (ID No. 15120), located 26 km north of the study site. The data were further arranged by including only the time period between 10 am-6 pm for each day. Because long-term historical data was not available for Haaren Station, the next closest wind station (40 km southeast), Arolsen-Volkhardinghausen (ID No. 197), was chosen to extract the wind data for the seed dispersal periods between October 2008 and February 2012. The precipitation data from Brilon-Thülen station (ID No. 6264) (4 km to the study site) was used to remove the effect of high relative humidity by subtracting the hours with precipitation from the corresponding wind data. The dispersal and wind data were analyzed in 18 angle classes each with 20° (18×20° = 360°). The patterns of pollen and seed dispersal direction and wind direction were assessed using ORIANA 4.02 (Kovach Computing Services, Pentraeth, Wales, UK) by constructing circular histograms, calculating mean directions, and computing circular correlations between dispersal directions and wind directions. Since the wind directions were also recorded as azimuth (0° North), there was no need for further conversion to make pairwise comparisons. To determine the effect of the frequency of strong winds on LDD, linear relationships between dispersal distances and wind speeds in the same angle classes were assessed using paired *t*-test for sample means. Pearson’s correlation coefficients and their *p*-values were calculated using (1) the means of the dispersal distances and wind speeds, and (2) the cumulative dispersal distances and wind speeds in each angle class.

## Results

### Population genetics parameters

The A_R_ was higher in the native adult trees (6.61) than in the cultivated adults (4.69), and compared to the native adults, it was slightly lower in the seedlings (6.47) and in the seeds (6.19) (Table 1). The F_IS_ level of the cultivated adults (0.012) was slightly higher than that of the native adults (0.006) (Table 1). Compared to the native adults, the seedlings had higher F_IS_ (0.021) while the seeds had lower (0.002) (Table 1). All pairwise F_ST_ values of genetic differentiation were highly significant (*p* < 0.001), and showed that the offspring were differentiated from the cultivated roadside trees (0.062 and 0.059, respectively; Table 2), but not from the native adults (0.004 and 0.005, respectively; Table 2). There were no large allele drop-out or stuttering detected in any loci. However, there were mother-offspring mismatches detected, particularly in FEMSATL8 and Fp104136 loci. The estimated mean null allele rates across loci for the adults and offspring were between 0.11 and 0.16 (Table 1).

**Table 1:** Genetic diversity parameters for the adults and offspring.

	N	A	A_R_	A_e_	H_o_	H_e_	F_IS_	NR
**Cultivated**	58	4.692	4.690	2.172	0.520	0.453	0.012	0.159
**Native**	210	8.462	6.610	3.576	0.485	0.488	0.006	0.109
**Seedlings**	247	8.308	6.470	3.223	0.457	0.483	0.021	0.136
**Seeds**	500	9.460	6.190	3.288	0.464	0.472	0.002	0.115

N, number of individuals; A, mean number of alleles; A_R_, allelic richness corrected for sample size; A_e_, effective number of alleles; H_o_, observed heterozygosity; H_e_, expected heterozygosity; F_IS_, sample mean inbreeding coefficient (Avg Fi); NR, mean null allele rates.

**Table 2:** F_ST_ pairwise genetic differentiations (below diagonal) and their significance (above diagonal) for the adults and offspring.

	Cultivated	Native	Seedlings	Seeds
**Cultivated**	0	***	***	***
**Native**	0.053	0	***	***
**Seedlings**	0.062	0.004	0	***
**Seeds**	0.059	0.005	0.006	0

*p* values were determined using 9999 permutations

*** *p* < 0.001

### Parentage analysis

#### Parentage assignments and mating model estimations

Total exclusion probabilities for both parentage analyses were high (0.999) in CERVUS. Thirteen loci provided the mean heterozygosity of 0.49, and the mean numbers of alleles per locus were 10.3 for paternity and 9.5 for parent pair analyses. Using Δ, unique candidate fathers were confidently assigned to 332 (66.4%) seeds, and among them 200 (62.1%) were strictly confident. No mismatching loci were detected in 228 (70.8%) of the assignments. Twenty paternal assignments were not statistically confident enough even though the candidate fathers had no mismatching loci with the mother and seed. In the case of the seedlings, the number of confidently assigned unique parent pairs was 121 (49%). The level of confidence was strict for 41 (17%) assignments. The overall number of trio loci with no mismatch was 110 in the parent pair analysis. Sixty-seven (27%) single parents were confidently assigned and among them 28 were strictly confident.

**Fig 2:**
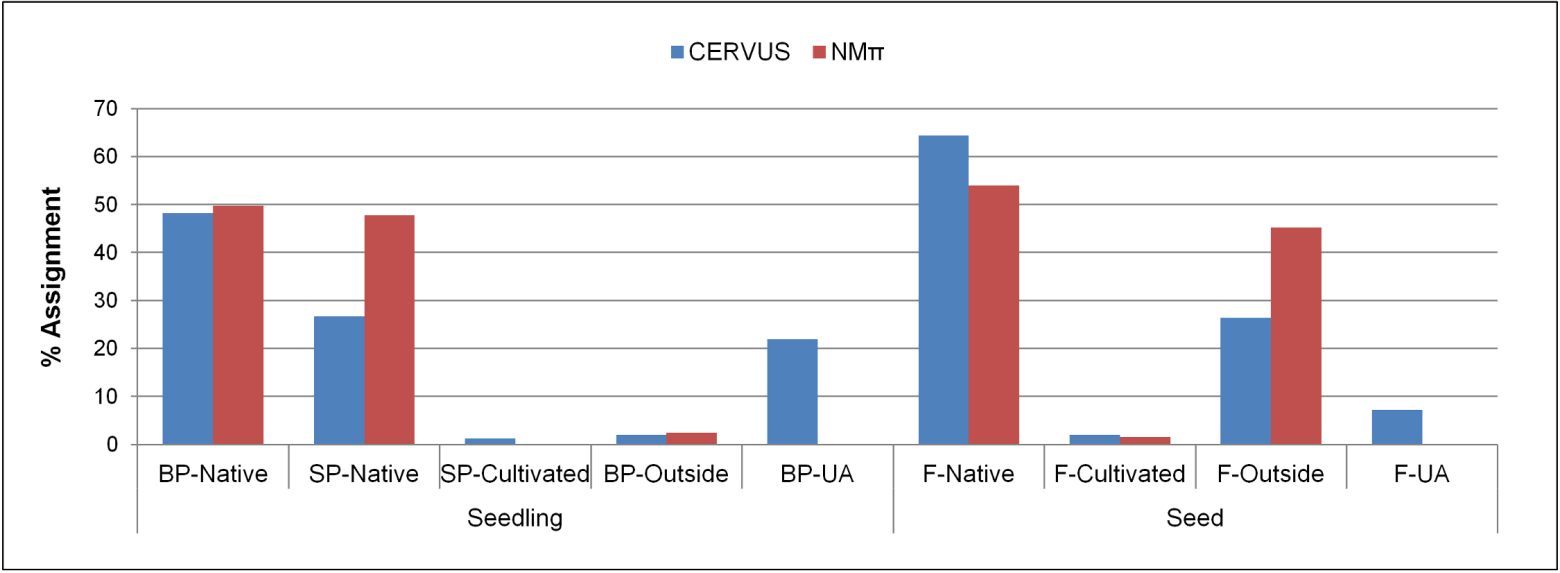
Breakdown of the parentage assignments (%). BP, both parents; SP, single parent; UA, unassigned; F, father.

The paternity and parent pair analyses revealed 64.4% and 74.9% of gene flow from the native trees while 2% and 1.2% from the cultivated trees, respectively (Fig 2). Although the trio LOD and Δ scores were positive, 7.2% of the paternity and 21.9% of the parent pair assignments were not confident enough (Fig 2). Respectively, 26.4% and 2% of the paternity and parent pair assignments presented negative LOD scores which are treated as putative immigrants (Fig 2). The overall selfing rate was very low (0.4%).

The AIC model comparisons of the applied neighbourhood models showed that the two- parameter exponential-power kernel with directionality (anisotropy) is the best model for the seed and pollen dispersal in the study site (S3 Table). The estimated mating model parameters and the standard errors using the best-fitting model were given in Table 3. According to the model estimations, pollen dispersal kernel follows a fat-tailed distribution (*bp* = 0.269, b < 1) while seed dispersal kernel rather follows a normal distribution (*bs* = 2.085, b = 2) (Table 3). The estimated frequency of seed and pollen immigrants were 3% and 45%, respectively, and the frequency of self-fertilization was 0 (Table 3).

**Table 3:** Mating model parameters estimated by the best-fitting neighbourhood model.

Parameter	*s*	*ms*	*mp*	*1/ds*	*1/dp*	*bs*	*bp*	*ks*	*kp*	*as*	*ap*
**Estimate**	0	0.032	0.455	0.026	0.002	2.085	0.269	0.934	1.099	0.155	0.076
**Std. Error**	-	0.015	0.020	0.002	0.001	0.437	0.065	0.125	0.116	0.021	0.015

*s*, frequency of self-fertilization; *ms/mp*, frequency of seed and pollen immigration from surrounding sources; *ds/dp*, mean distance of seed and pollen dispersal; *bs/bp*, shape of seed and pollen dispersal kernels; *ks/kp*, rate of directionality (anisotropy) of seed and pollen dispersal; *as/ap*, prevailing direction (azimuth) of seed and pollen dispersal (% 2π).

Based on the best-fitting model, the parental profiles of the progenies were assessed according to the posterior probabilities of the two most-likely parentages inferred for each progeny (Fig 2). According to the parental profile, 123 (49.8%) seedlings have both their parents inside of the sampled stand while 118 (47.8%) seedlings have only mothers in the stand, and all of these parents are the native trees (Fig 2). On the other hand, 270 (54%) seeds were sired by the local native fathers, while only 4 (1.6%) of them were sired by the fathers from the cultivated alley trees (Fig 2).

### Realized and potential dispersal distances

**Fig 3:**
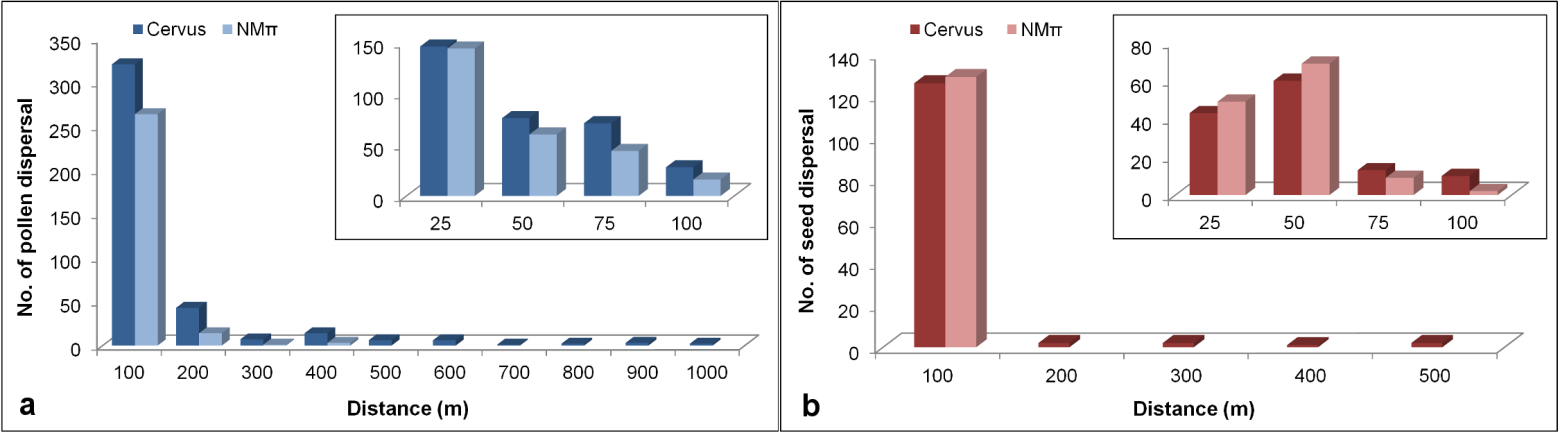
**Frequency distributions of pollen (a) and seed (b) dispersal distances.** Lower left shows dispersal events in 100 m distance classes; Upper right shows close-up on short distance dispersal events up to 100 m.

The frequency distribution graphs of pollen and seed dispersal distances were constructed based on the dispersal events identified using two approaches to parentage analysis (Fig 3). A total of 405 (295 paternity + 110 parent pair) CERVUS- assigned and 282 NMπ- inferred pollen dispersal events were used to create the pollen dispersal histograms (Fig 3A). According to the CERVUS assignments, the pollination distances ranged from 0 to 939.26 m with a mean of 90.48 m (median = 43.86 m) while the estimated pollination distances by the neighbourhood model ranged from 2.24 m to 339.75 m with a mean of 40.47 m (median = 23.71 m) (Fig 3A). The seed dispersal histograms were constructed with 133 CERVUS- determined (110 parent pair and 23 single seed parent) and 129 NMπ- inferred seed dispersal events (Fig 3A). The range of seed dispersal distance detected using CERVUS was between 1.83 m and 473.53 m with the mean distance of 48.60 m (median = 30.77 m) (Fig 3B). NMπ- computed seed dispersal distances were between 1.83 m and 89.61 m with the mean distance of 30.37 m (median = 28.81 m) (Fig 3B). The majority of the pollen dispersal detected by CERVUS (79%) and NMπ (94%) occurred up to 100 m (Fig 3B), similar to the seed dispersal detected by CERVUS (95%; Fig 3B). There was no seed dispersal detected beyond 100 m (Fig 3B) with NMπ while the CERVUS- identified seed dispersal beyond 100 m was 5.3% (Fig 3B). The identified pollen dispersal beyond 100 m was 21% with CERVUS and 6% with NMπ (Fig 3B).

**Fig 4:**
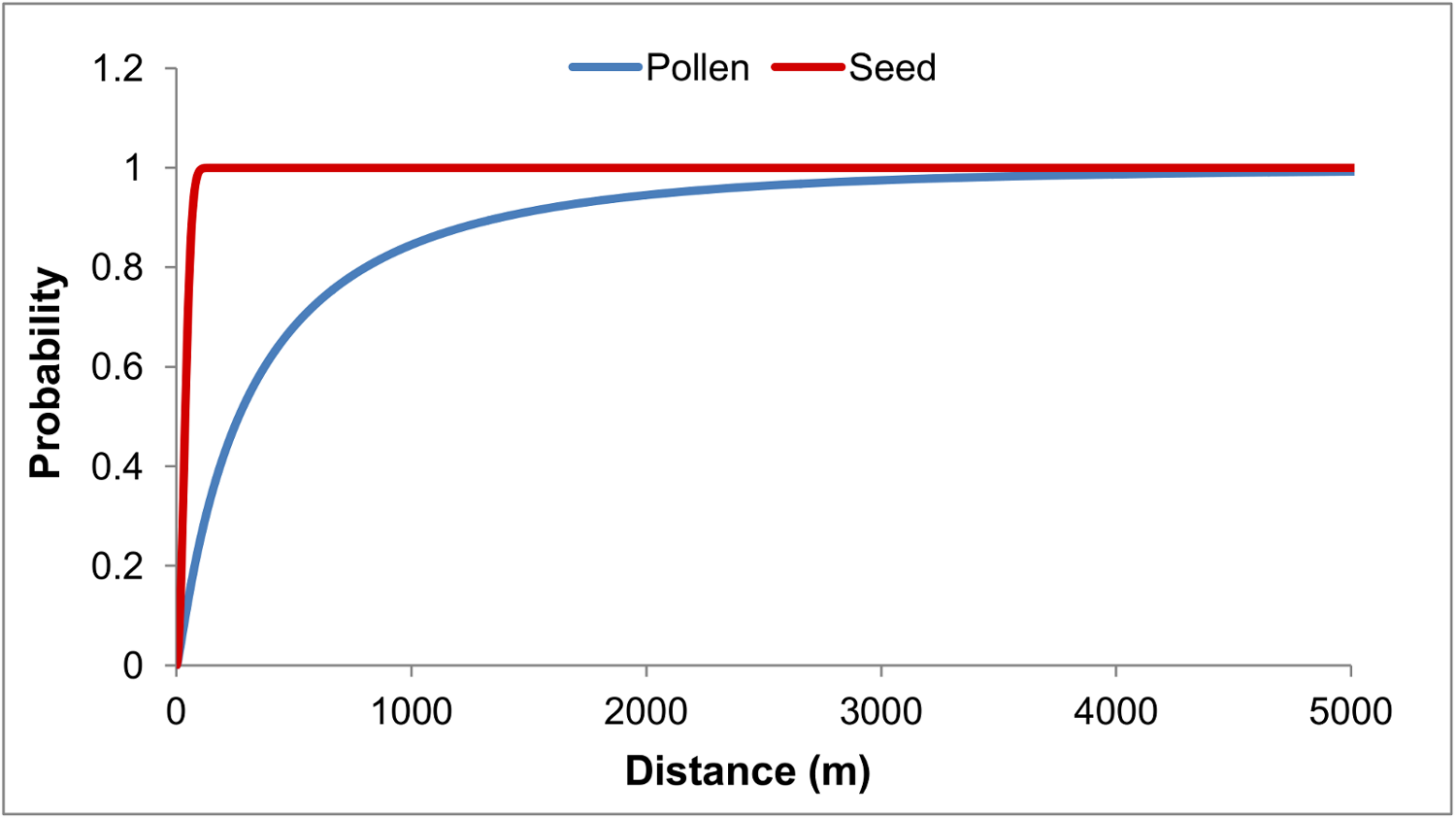
Cumulative probability distributions for the maximum likely dispersal kernel parameters.

The NMπ- estimated dispersal kernel parameters *ds*, *bs*, *dp* and *bp*, and the approximate 95% confidence interval around them were given in Table 4. Based on the fitted kernel function, the estimated average distances of seed (*ds*) and pollen (*dp*) were 39 m and 571 m, respectively (Table 4). According to the cumulative probability distributions obtained for the maximum likely dispersal kernel parameters (Fig 4), 50% of the seeds travel not further than 37 m and less than 1% seeds travel further than 94 m. Compared to the seeds, 50% of pollen disperses up to 266 m while 10% pollen disperses further than 1 380 m, and 1% pollen can reach distances as far as 4 478 m or more from the source plant.

**Table 4:** The approximate 95% confidence interval around dispersal kernel parameters.

Parameter	Estimate	Q [2.5%]	Q [97.5%]
***ds***	39.1	33.9	46.2
***bs***	2.085	1.016	3.154
***dp***	571.0	201.6	Infinity
***bp***	0.269	0.110	0.428

### Dispersal patterns versus wind patterns

Pearson’s correlation coefficients (*r*) and their significances were calculated for linear associations between dispersal distances and wind speeds in the same angle classes, in the corresponding dispersal period (Table 5). Correlations were assessed by taking the mean and the cumulative dispersal distances and wind speeds, respectively (Table 5). Both correlations were found to be moderate and negative (-0.332 and -0.374, respectively) between pollen dispersal distance and wind speed. A positive but weaker correlation was calculated between seed dispersal distance and wind speed (0.212). However, the correlation was much stronger (0.715) when the cumulative distances and speeds were taken into account. All correlations were highly significant (*p* < 0.01).

**Table 5:** Linear correlations between dispersal distances (DD) and wind speed (WS) in the same angle classes.

Dispersal Period	Mean DD and WS		Cumulative DD and WS	
	*Pearson’s r*	*p value*	*Pearson’s r*	*p value*
**Pollen**	-0.374	0.0047	-0.332	0.0009
**Seed**	0.212	0.0004	0.715	0.0073

**Fig 5:**
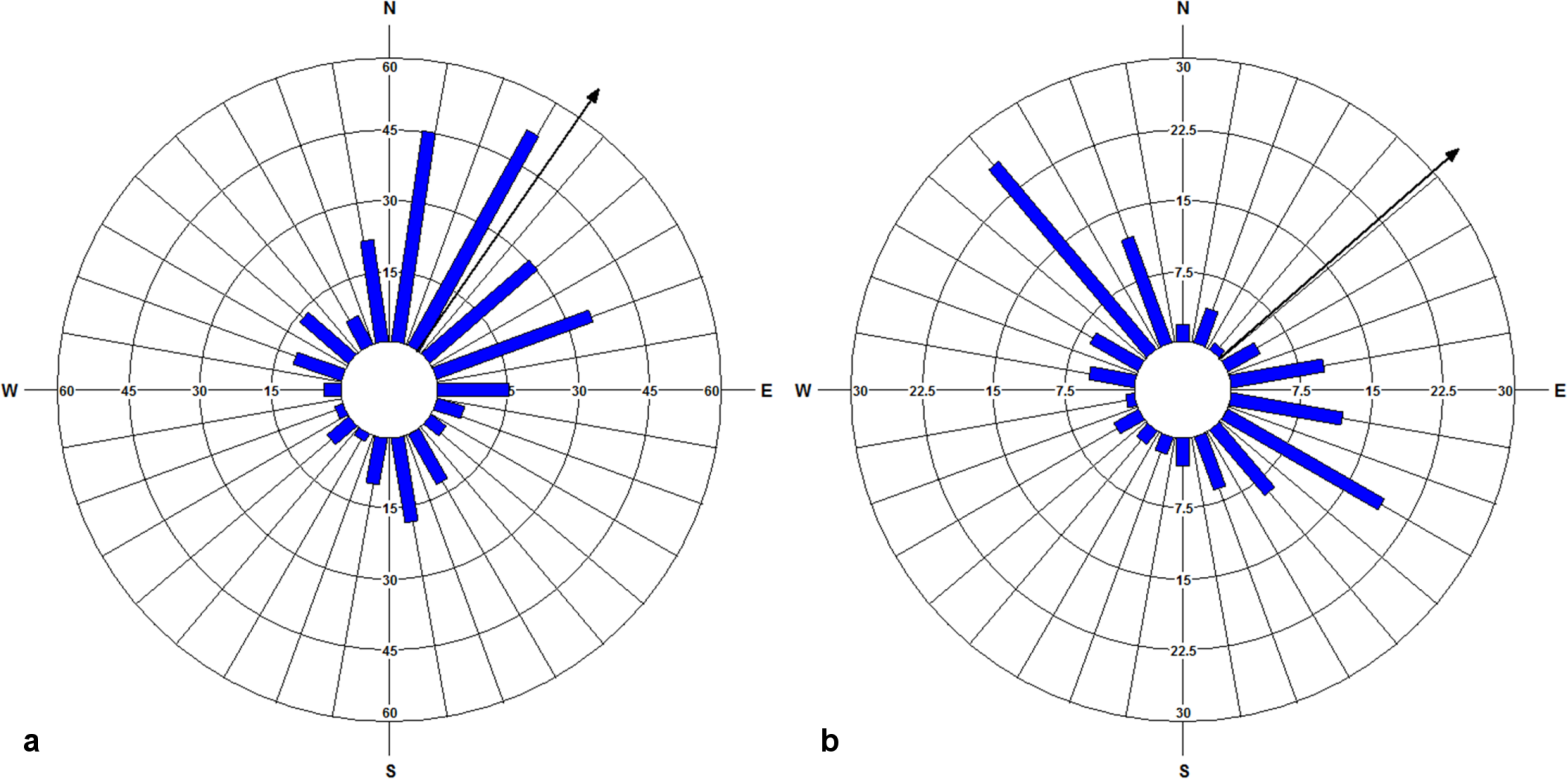
Circular histograms for pollen dispersal period in 20° angle classes. Blue bars represent (a) mean pollen dispersal directions and (b) mean wind directions; Black arrows show mean directions.

**Fig 6:**
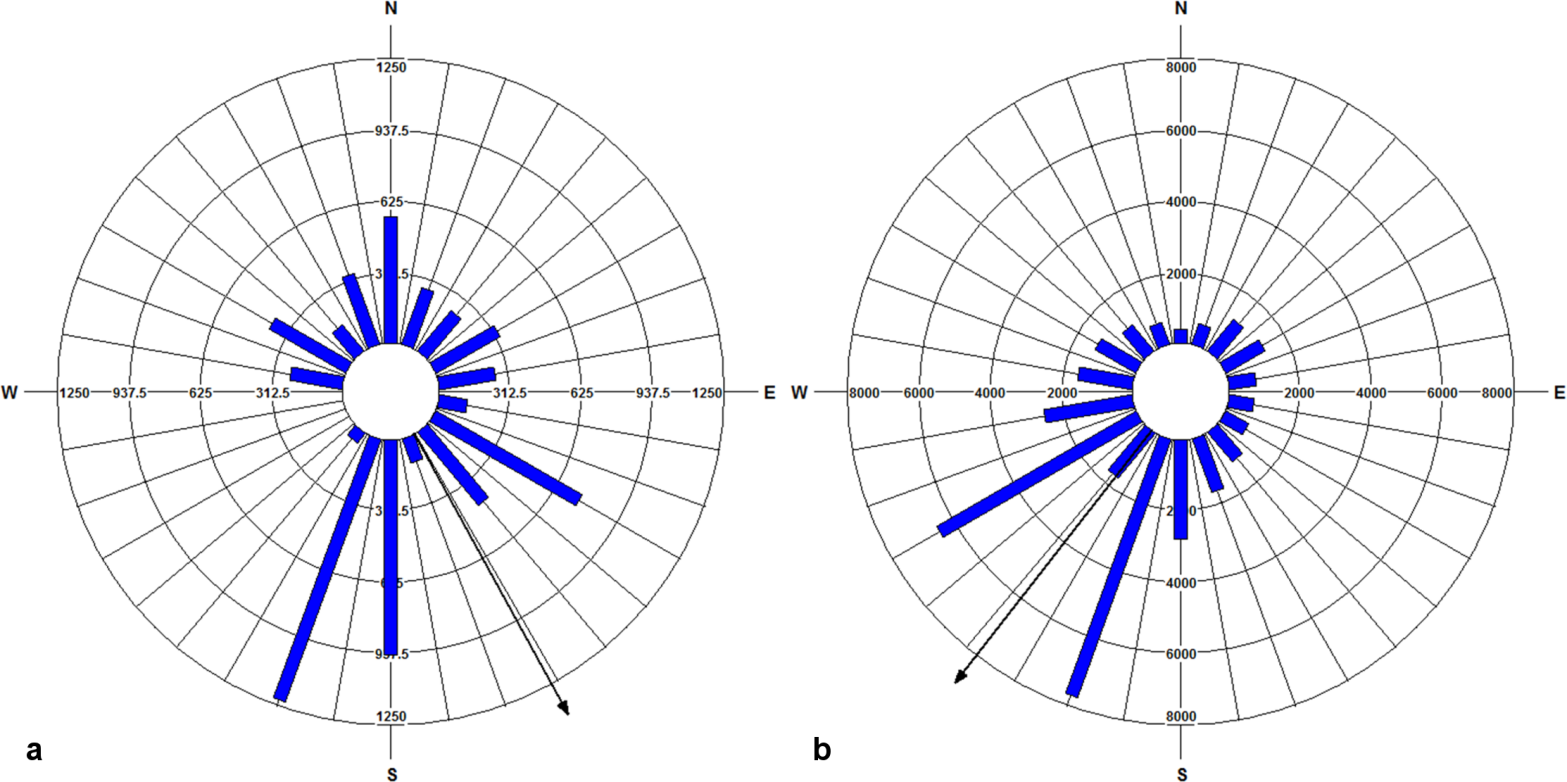
Circular histograms of seed dispersal period in 20° angle classes. Blue bars depict (a) mean seed dispersal directions weighted by dispersal distances and (b) mean wind directions weighted by wind speed; Black arrows show mean directions.

The realized pollen and seed dispersals and wind patterns during the pollination and seed dispersal periods were graphed using circular histograms (Figs 5 and 6). The pollen dispersals occurred in all directions in accordance with the wind directions (Fig 5A and 5B). Mean pollination direction was 34.78° (Fig 5A) and mean wind direction was 48.71° (Fig 5B), both towards the northeast. The circular correlation between pollination and wind direction was calculated as 0.999 (*p* < 0.05). The estimated prevailing direction of pollen dispersal (*ap*) with the fitted model was 27.36° (*ap* = 0.076 in % 2π; Table 3), also towards the northeast.

Circular-linear histograms were constructed for seed dispersal and wind patterns for four dispersal periods between October 2008 and February 2012, by incorporating the linear components (dispersal distance and wind speed) into the angles (dispersal direction and wind direction, respectively) (Fig 6B). The seed dispersal events were identified in all angle classes except for 240° and 260°, the southwest directions (Fig 6A), while the wind blew in all directions (Fig 6B). The mean dispersal direction weighted by dispersal distance was calculated as 145.32° towards the southeast (Fig 6A). The mean wind direction, weighted by speed, was calculated as 217.88° towards the southwest (Fig 6A). The circular correlation between the seed dispersal direction and wind direction was 0.682 (*p* < 0.05). However, when only direction was taken into account, the mean seed dispersal direction was towards the northeast (59.84°), which was similar to the neighbourhood model estimations for the mean prevailing seed dispersal direction (55.8° = 0.155 in % 2π; Table 3).

## Discussion

Small and isolated populations may have limited genetic variation due to low genetic diversity, which may jeopardize their adaptive potential to various threats such as EIDs. However, common ash populations in central Europe often show high genetic diversity and low genetic differentiation due to LDD of ash seed and pollen, which may reduce the risk of small population size and increased isolation [75]. Similarly, we observed high genetic diversity in our study site in Rösenbeck. The mean H_e_ across the 3 genomic SSR loci was very high: 0.89 for the native adults and 0.87 for the seedlings and seeds, although lower, 0.72, for the cultivated adults. 10 EST-SSR loci showed much lower mean H_e_, which was 0.37 for the cultivated and native adults and for the seedlings, and 0.35 for the seeds. Lower polymorphisms are well known in EST-SSRs because they are located within expressed genes and therefore are more conserved compared to genomic SSRs [76]. The mean H_e_ calculated for all 13 SSRs was higher in the native adults (0.49) and in the natural regeneration (0.48) compared to the cultivated trees (0.45) (Table 1). Using 6 neutral SSRs, high levels of gene diversities in *F*. *excelsior* adults (H_e_ = 0.767–0.818) and progenies (H_e_ = 0.777–0.805) have previously been reported in two sites in Ireland [77]. In the same study, planted adults were genetically not much differentiated from the native adults (F_ST_ = 0.013 in Clonee and F_ST_ = 0.014 in Kildalkey). Our pairwise F_ST_ values showed higher genetic differentiation between cultivated and native trees (0.053; Table 2). This result is in accordance with the potential clonal origin of the cultivated trees.

High levels of homozygosity have been previously reported in *F*. *excelsior* populations, reflecting a likely mix of Wahlund effect, null alleles, inbreeding and large allele drop-out [4,38,78–80]. Recently, a lower level of mean F_IS_ (0.067) in ash populations in Northern Ireland has been reported [38]. Thomasset et al. [77] has also shown low levels of mean F_IS_ in two native ash populations (F_IS_ = 0.053 and 0.057), but higher mean F_IS_ in their progenies (F_IS_ = 0.131 and 0.122). In our study, the mean inbreeding coefficients for the adults and the offspring were much lower. The F_IS_ of the native adults was 0.006, lower than that of the seedlings (F_IS_ = 0.021) but higher than that of the seeds (F_IS_ = 0.002) (Table 1). The higher F_IS_ level of the seedlings may result from the sampling effect, which was along the three transect lines covering the different parts of the sampling site. Based on the same Bayesian procedure we used, Tollefsrud et al. [75] also reported very low inbreeding levels in 42 natural ash populations, even at the northern range margins, and attributed the differences in F_IS_ values among studies to the differences in SSR loci and methods used.

Categorical parentage approaches are powerful in detecting gene flow when microsatellite markers are highly polymorphic and sampling is efficient [81]. However, they may be inefficient to discriminate local and background pollen and seed sources if exclusion probabilities of genetic markers are not sufficient [82]. Unassigned parentages often occur due to both unsampled parents and sampled but not assigned local parents. Additionally, microsatellites are often prone to genotyping errors including null alleles [83] and mutations [84], and cryptic gene flow may lead to false-positive assignments [85]. This bias is expected to be lowered using mating models since they estimate gene dispersal parameters from probability models and can account for observed frequencies of multilocus genotypes in offspring, rather than from directly inferred parent-offspring pairs [86]. The empirical distributions of effective dispersal distances may also be biased by spatial distribution of individuals in a population [87]. The neighbourhood model can simultaneously estimate the rate of immigrants and mating parameters from given allele frequencies when accounting for the spatial arrangement of individuals and other available information on reproductive success [82]. For that reason, we applied two approaches to investigate seed and pollen dispersal patterns in our study.

The overall successes of the both parentage analyses using CERVUS were high in our study. Not only because using 13 loci with no missing allelic information provided us high exclusion probabilities (0.999), but also because the exhaustive sampling scheme of the adult trees played a role in increasing the chances of sampling the true parents. We also allowed mismatches (1%) in the parentage analyses to increase the overall success rate [69]. We confidently assigned 66.4% of the seeds to their unique fathers, 48.2% of the seedlings to their unique parent pairs and an additional 27.9% of the seedlings to their single parents (Fig 2). These assignment rates are high compared to the two most recent studies reporting on gene flow in ash using the same parentage analysis software. Taking Δ as a confidence measure and assuming 10% of the potential parents sampled, Thomasset et al. [77] assigned 11.5–15% of the seeds to their unique fathers at 80% confidence level using 6 microsatellite loci. However, higher rates of sampling potential parents (95%) with 7 loci resulted in higher assignment rates of the ash saplings to their parent pairs (44.1%) in Northern Ireland, at least in one site [38]. Therefore, the success rate of the assignments is highly influenced by the number of adults sampled, marker loci used and confidence level chosen. However, CERVUS left respectively 33.6% of the paternities and 23.9% of the parent pairs unassigned (Fig 2) and the estimated mean null allele rate was 13% overall. The neighbourhood model estimated 3.2% and 45.5% of seed and pollen immigration from outside of the study site, respectively (Fig 2; Table 3). In case of seed immigration, this result is consistent with the CERVUS assignments which had negative LOD scores and were treated as putatively from outside (2%; Fig 2). The neighbourhood model inferred that mothers of the remaining seedlings (21.9%) unassigned by CERVUS were also sourced from the local native trees (Fig 2). The NMπ estimation for pollen immigration was higher (45.5%) compared to the CERVUS estimation (33.6%), which may indicate the presence of false-positive assignments among the assigned candidate fathers with 80% confidence (Fig 2). On the other hand, the model used here is spatially explicit and assumes that male mating success decreases exponentially with increasing distance from mother and seedling [34,35]. However, distance alone often explains a relatively moderate portion of the variation in mating patterns [37], and model predictions can be improved with additional parameters influencing individual mating success [72]. Using the neighbourhood model approach based on a 3000 m radius neighbourhood, Bacles et al. [4] reported higher pollen immigration (53%) in a severely fragmented population of *F*. *excelsior* in Scotland. When including the reproductive success parameters such as plant size and flowering intensity to the neighbourhood model, Goto et al. [88] estimated 52.5% of pollen and 31.5% of seed immigration in *F*. *mandschurica* in a 10.5 ha plot in Japan. However, since the estimated immigration rates are sensitive to the defined neighbourhood size and the selection gradients influencing male mating success, model comparisons still remain as a challenge [72]. The results overall indicate that ash propagule, particularly pollen, has a great capacity for LDD. This is consistent with the complete sampling scheme of the study site and the demography of ash in a radius of 5 km around the study site. The cumulative probability distribution of pollen dispersal distances based on the best-fitting dispersal kernel (Fig 4) also provides a good support for the species’ LDD potential.

Molecular markers provide enough information to assign parentage to offspring. However, reliable identification of pollen and seed dispersal distances requires additional information regarding the gender of the parental trees. This is relatively easy for the pollen dispersals as long as prior information about the genotypes of mother trees from which the seeds were collected is available. It presents a greater challenge to determine pollen and seed parents of established seedlings. Seed trapping can provide information about the genotype of maternal tissue [89,90] that can only be informative for seeds. The number of dispersed seeds, their survival and recovery rates via seed tagging are also shown to be very low [91]. Bacles et al. [5] calculated seed dispersal distances among fragmented populations of *F*. *excelsior* based on a prior assumption of the nearest, or the single parent identified being the maternal parent. However, this assumption may not always be true and therefore may cause an erroneous conclusion such that seed dispersal distances are always shorter than pollen dispersal distances [37]. Additionally, chloroplast haplotypes only provide limited help to identify the seed trees in ash [38] because their polymorphism levels at regional scales are very low, and a single haplotype dominates in most regions [39]. Two-year macroscopic observations of ash flowers in the study site made the gender identification of the adult trees possible in most of the cases: 89% of assigned paternities and 91% of the assigned parent pairs were confirmed by our field observations. We also accounted for the genders of the adult trees in the neighbourhood model estimations and the inferred parentages. This provided more accurate estimations of the dispersal patterns in the study site.

Plant improvement is based on the breeding of plants with desirable traits; however, gene flow from domesticated species cannot be regarded as universally beneficial [92]. In some cases, interpopulation crosses result in lowered fitness in the next generations that can be attributed to the disruption of local adaptation [93]. For this reason, pollen contamination via immigration of genes from domesticated populations to natural populations, and gene conservation reserves particularly, raises concern [10]. *F*. *excelsior* is one of the tree species silviculturally managed in the last 30–40 years in Europe, mostly due to its high quality wood [94]. It is also extensively used in urban landscape planting because of the aesthetic characteristics desired from urban trees such as autumn colour, attractive bark and flowers [95]. Planting density in ash varies across Europe from 500 to 10 000 trees per ha [20]. According to the National Forest Inventory, from 2002 to 2012, the area covered with ash in Germany increased by 17.4% (250 000 ha), which represents 2.4% of the total forest area [96]. Accordingly, measuring gene flow in the landscapes can help us infer the potential impacts of plantation practices. Using two approaches to parentage analysis, we estimated very limited gene flow from the cultivated trees to the native trees (1.2–2%; Fig 2) in our study site. The majority of the gene flow occurred in the landscape among the native trees (Fig 2). This result may not be surprising due to the small size of the plantation compared to the native stand. In the two ash sites in Ireland, paternity assignments of the seeds revealed similar mating success of introduced and native trees and the offspring of native and planted trees were sired by native and planted trees [77]. In the same study, partial temporal isolation between planted and native trees better explained the structure of the pollen clouds. However, that was not an indication in our case. Flower observations were conducted twice (one per year) in our study site during the peak flowering time for both planted and native trees. In 2014, the year when the seeds were collected, we observed the flowers between the 11^th^ and 17^th^ of April. The German Weather Service also recorded first ash pollen release in the region on the 15^th^ of April in 2014 (Station ID: 9247) (Available from: ftp://ftp-cdc.dwd.de/pub/CDC/observations_germany/phenology/annual_reporters/wild/historical/PH_Jahresmelder_Wildwachsende_Pflanze_Esche_1951_2015_hist.txt). The small stand size of the cultivated trees compared to the native stand, their relative distance from the native mothers and the local wind direction may have affected the pollen clouds in our study site. On the other hand, Steinitz et al. [97] estimated higher gene flow levels from *Pinus halepensis* plantations to two conspecific native populations; the more isolated population had a lower gene flow level (22%) than the less isolated one (49%). The differences in seed and pollen dispersal results among different sites are highly dependent on phenological synchrony and relative fecundity of trees [33,56], spatial proximity of populations and individuals [98], size of source and sink populations [99], topography of sites [100], wind direction and speed [101] as well as sampling scheme [32], intrinsic characteristics of plant diaspore such as size and shape [102] and on the method of choice [103].

Short distance dispersal (SDD) mainly shapes the local distribution of genotypes [1]. LDD of pollen and seed are regarded as critical in understanding many processes such as habitat fragmentation, genetic differentiation of populations, evolutionary responses to climate change and biological invasions [104]. The ability of trees for LDD is suggested to possibly promote their adaptive evolution in new environments through increasing genetic variation for fitness [48]. Seed dispersal is of special interest because a diploid seed carries twice the genetic material compared to a pollen grain [7], and especially because it is crucial for the ability of species to continuously colonize suitable sites in the mosaic of different microenvironments across managed and unmanaged forests and landscapes. In plants, pollen and seed dispersal typically follow a fat-tailed or leptokurtic distribution, indicating frequent nearest-neighbour mating along with rare LDD [105]. In our study, the estimated pollen dispersal kernel also followed a fat-tailed distribution (*bp* = 0.269, b < 1; Table 3), with an average pollen dispersal distance of 571 m (Table 4). However, the seed dispersal distribution followed the Gaussian or a platykurtic kernel (*bs* = 2.085, b = 2; Table 3), indicating that the mean seed density declined more rapidly beyond the mean dispersal distance of 39 m (Table 4). Based on the neighbourhood approach, Bacles et al. [4] reported a fat-tailed pollen dispersal kernel with a mean dispersal distance of 328 m in a severely fragmented population of *F*. *excelsior* in Scotland. In the same population, however, the predicted seed dispersal distribution was also fat-tailed, suggesting that the seed dispersal may also spread over tens of kilometres, and the authors concluded that deforested landscapes favors LDD of seed due to the increased likelihood of winged-seed to be uplifted [5]. However, these results, including ours, were based on a single year and lack of inter-annual replications in the study sites and may present a limitation to the estimations because spatial and mating system parameters show significant temporal variation and the regeneration of natural stands generally takes years [106]. Based on CERVUS assignments, we detected 50% of the effective pollen dispersed more than 44 m from the source trees, and 5% beyond 467 m, up to 939 m (Fig 3A) whereas 50% of the seeds were dispersed within 31 m from the source, and 5% beyond 108 m, up to 473 m (Fig 3B). However, NMπ- computed distributions of the dispersal distances in the study site were both shorter, particularly for the seed dispersal distances (Fig 3A and 3B). The difference between the observed effective pollen dispersal distributions and the estimated dispersal kernels is due to their definitions: they represent backward and forward dispersal kernels, respectively, and thus different notions to describe the dispersal process [107]. On the other hand, LDD in different *F*. *excelsior* populations has been previously reported for both pollen [6,77] and seed [38] using parentage analysis approaches. The results overall suggest that gene flow in ash can occur in a wide range of distance, from tens or hundreds of meters to thousands. This is promising for the future existence of the species in our ecosystems considering ongoing habitat fragmentation, climate change and ash dieback. However, it also complicates the *in situ* gene conservation practices of the species.

Particularly for wind-mediated gene flow, available weather data could help to identify the potential patterns of pollen and seed dispersals in landscapes, and to better understand the meteorological factors driving pollen and seed emission and spread [48]. Using the previous knowledge of the weather conditions favouring propagule release and dispersal could also provide more accuracy in timing of these events. Among these, dry and windy weather conditions are stated to promote LDD of seeds [40–43]. Similarly, clear warm days with low relative humidity, especially from midday to late afternoon (10 am-6 pm), are also reported as most favourable for pollen release [44–46]. Our results showed that dry and windy weather conditions influenced the pollen and seed dispersal patterns in our study site. There was a significant positive correlation between the dispersal distances of the seeds and strong winds (0.715; *p* < 0.01; Table 5), indicating that frequent strong winds favoured LDD of seeds. Heydel et al. [101] pointed out a mainly positive relationship between horizontal wind speed and LDD in both forests and open landscapes, and showed the increase in the strength of the relationship with seed terminal velocity (V_term_) during the fall. According to their results, turbulence is positively correlated to LDD for the species with Vterm ≥ 0.8 m/s and the effect of LDD changes from positive (Vterm = 3.2 m/s) to negative (Vterm ≤ 0.4 m/s). This is consistent with our results when considering the medium V_term_ of *F*. *excelsior* (1.58 m/s). However, pollen dispersal distances were negatively correlated with the wind speeds (Table 5). This may not be surprising because maximum turbulence above the canopy often occurs in the afternoon when maximum pollen release also happens [108], and mechanical turbulence dominating at high wind speeds often results in downdrafts [109]. Other factors including propagule release height, vegetation type, topography, and landscape terrain are also previously mentioned as likely to influence LDD of plant propagules [101]. The ability of ash trees to maintain part of their fruits long after leaf shed may favour LDD of seeds that are only released during heavy autumn or winter storms. However, investigating the relative effects of these factors on dispersal events is beyond the scope of our study. In the study site, both pollen and seeds were dispersed in almost all directions in accordance with the actual wind directions (Figs 5 and 6). The mean direction of pollen dispersal had a good agreement with the mean wind direction, both towards the northeast (Fig 5). Similarly, the model estimations for the mean distributions of pollen and seed dispersal also indicated directionality, both towards the northeast (Table 3), in agreement with the prevailing wind direction in the study site. This may indicate unequal mating success of the males along with restricted dispersal in the study site, predominantly driven by the prevailing winds. Nonetheless, the mean direction of seed dispersal was towards the southeast while the mean wind direction was towards the southwest, when incorporating the dispersal distances and the wind speeds (Fig 6). This could be because of the unidentified LDD of seed in the angle classes towards the southwest direction, pulling the mean towards the southeast direction. Furthermore, the wind data used for the seed dispersal period was from a long-term data set, covering four dispersal seasons in between October and February. Nevertheless, overall findings were informative to understand the effect of dry and windy weather conditions on the propagule dispersals in our study site.

*H*. *fraxineus* has spread across most of its distribution area in Europe within the last couple of decades, and is threatening *F*. *excelsior*’s existence and its associated ecosystem [19,110]. The emerald ash borer (EAB), *Agrilus planipennis* (Coleoptera, Buprestidae), on the other hand, has been predicted to cross the western border of Russia soon [111]. The fungus-weakened ash is expected to be more vulnerable to EAB attacks [112]. High standing genetic variation together with the ability for LDD of *F*. *excelsior* is promising for the species’ existence in the European forest ecosystems and for its adaptive potential. Recent reports on the presence of genetic variation in dieback tolerance among ash trees [29,113] suggest the species’ ability to recover from ongoing epidemic dieback [27]. However, the standing genetic variation will greatly depend on the individuals’ survival and reproduction, and the speed of population recovery will largely depend on the extent of pollen- and seed- mediated gene flow. Therefore, it presents importance to estimate gene flow patterns of ash populations in European landscapes based on a good experimental design and on combined analytical approaches to provide reliable information for the conservation management of ash.

## Conclusion

Our results indicated an effective landscape process of gene flow in ash including the LDD of pollen and seed and the effect of the wind conditions on the dispersal patterns. In our study, the cultivated roadside trees in the landscape only provided limited gene flow into the old growth forest. However, our results still suggest that gene conservation stands should be planned carefully in ash, considering the LDD of ash propagule. A maximum of 100 m distance among trees may be recommendable for effective enrichment plantings in order to facilitate gene flow from healthy to unhealthy individuals. However, our results are based on a single small stand of *F*. *excelsior*, and the relative distributions of individuals, sampling design, the method of choice and local wind conditions seem to influence the estimated seed and pollen dispersal patterns in the study site. A combination of different analytical approaches to parentage analysis can provide a better resolution to gene flow studies. Available wind data from weather stations in proximity can be utilized for planning purposes.

## Supporting information

S1 Table Geographical coordinates, gender, seed set and genotypes of the cultivated and native adults and offspring. (XLSX)

S2 Table Meteorological data used in the study. (XLSX)

S3 Table Model comparisons. (XLSX)
